# Understanding the integration of artificial intelligence in healthcare organisations and systems through the NASSS framework: a qualitative study in a leading Canadian academic centre

**DOI:** 10.1186/s12913-024-11112-x

**Published:** 2024-06-03

**Authors:** Hassane Alami, Pascale Lehoux, Chrysanthi Papoutsi, Sara E. Shaw, Richard Fleet, Jean-Paul Fortin

**Affiliations:** 1 Department of Health Management, Evaluation and Policy, School of Public Health, University of Montreal, P.O. Box 6128, Branch Centre-Ville, Montreal, QC H3C 3J7 Canada; 2 Center for Public Health Research of the University of Montreal, Montreal, QC Canada; 3 Institute for Data Valorization (IVADO), Montreal, QC Canada; 4 Nuffield Department of Primary Care Health Sciences, University of Oxford, Oxford, UK; 5 Faculty of Medicine, Laval University, Quebec, QC Canada; 6 VITAM Research Centre on Sustainable Health, Faculty of Medicine, Laval University, Quebec, QC Canada

**Keywords:** Artificial intelligence, Digital health, Health organisation, Health system, Business models, Complexity, Evaluation, Implementation, Scale-up, Innovation adoption

## Abstract

**Background:**

Artificial intelligence (AI) technologies are expected to “revolutionise” healthcare. However, despite their promises, their integration within healthcare organisations and systems remains limited. The objective of this study is to explore and understand the systemic challenges and implications of their integration in a leading Canadian academic hospital.

**Methods:**

Semi-structured interviews were conducted with 29 stakeholders concerned by the integration of a large set of AI technologies within the organisation (e.g., managers, clinicians, researchers, patients, technology providers). Data were collected and analysed using the Non-Adoption, Abandonment, Scale-up, Spread, Sustainability (NASSS) framework.

**Results:**

Among enabling factors and conditions, our findings highlight: a supportive organisational culture and leadership leading to a coherent organisational innovation narrative; mutual trust and transparent communication between senior management and frontline teams; the presence of champions, translators, and boundary spanners for AI able to build bridges and trust; and the capacity to attract technical and clinical talents and expertise.

Constraints and barriers include: contrasting definitions of the value of AI technologies and ways to measure such value; lack of real-life and context-based evidence; varying patients’ digital and health literacy capacities; misalignments between organisational dynamics, clinical and administrative processes, infrastructures, and AI technologies; lack of funding mechanisms covering the implementation, adaptation, and expertise required; challenges arising from practice change, new expertise development, and professional identities; lack of official professional, reimbursement, and insurance guidelines; lack of pre- and post-market approval legal and governance frameworks; diversity of the business and financing models for AI technologies; and misalignments between investors’ priorities and the needs and expectations of healthcare organisations and systems.

**Conclusion:**

Thanks to the multidimensional NASSS framework, this study provides original insights and a detailed learning base for analysing AI technologies in healthcare from a thorough socio-technical perspective. Our findings highlight the importance of considering the complexity characterising healthcare organisations and systems in current efforts to introduce AI technologies within clinical routines. This study adds to the existing literature and can inform decision-making towards a judicious, responsible, and sustainable integration of these technologies in healthcare organisations and systems.

## Background

According to the Organisation for Economic Co-operation and Development (OECD), artificial intelligence (AI) refers to “a machine-based system that, for explicit or implicit objectives, infers, from the input it receives, how to generate outputs such as predictions, content, recommendations, or decisions that can influence physical or virtual environments. Different AI systems vary in their levels of autonomy and adaptiveness after deployment” [1]. Unlike conventional software, many AI systems indeed have learning capabilities and self-correcting error mechanisms that allow them to improve the accuracy of their results based on the feedback they receive [1, 2].

There are many application areas for AI in healthcare, for example: diagnosis, treatment, monitoring (e.g., chronic diseases), and patient compliance [3]. In certain experimental settings, AI technologies have been shown to be more effective than clinicians (e.g., diagnostic accuracy, more personalised diagnostics) [4–7]. Several have already been approved for clinical use in real-world care and services [8]. These technologies are seen as a lever for evidence-based clinical decision-making and practice and for value-based care and services [9–11]. Research indicates their potential to contribute to better monitoring, detection, and diagnosis of diseases, to the reduction of clinical risk, and to the discovery of new drugs and treatments [4, 9, 12–14]. The use of AI technologies could help to reduce diagnostic and therapeutic errors [2], contribute to the optimisation of clinicians’ work, and help reduce waiting times by reorganising clinical and administrative tasks, and supporting coordination [10, 14]. Many scholars also argue that AI technologies could contribute to reducing healthcare costs by decreasing hospital (re)admissions, medical visits, and treatments [14, 15].

A predominant and enthusiastic discourse in the academic literature and media reports is that AI technologies will revolutionise and radically change healthcare in the coming years [2, 16–18]. There is an explosion of AI offerings in the market [19]. In 2018, the global AI market in healthcare was valued at around US$1.4 billion and is expected to grow to US$17.8 billion by 2025 [14]. In North America, the market for AI in healthcare had exceeded US$1.15 billion by 2020 [14]. In this context, healthcare organisations and systems are increasingly being solicited (or even pressured) to integrate these technologies, even when evidence of real clinical added value is lacking and many social and ethical as well as adoption, routinisation, and practical issues remain to be clarified [16, 18]. According to Topol (2019), who reviewed healthcare workforce readiness for a digital future: “Despite all the promises of AI technology, there are formidable obstacles and pitfalls. The state of AI hype has far exceeded the state of AI science, especially when it pertains to validation and readiness for implementation in patient care” [4]. Liu et al. (2019) reported that few published studies on AI had results from real-world healthcare contexts [20]. These findings were corroborated during the COVID-19 pandemic [21–23]. Wynants et al. (2020) identified 232 AI models for prediction or diagnosis of COVID-19, none of which were appropriate for clinical use and only two showing potential for future clinical use [24]. Roberts et al. (2021) analysed 415 AI models for COVID-19 detection and concluded similarly [25].

This gap between the promise and reality of AI technologies in healthcare could be explained by the fact that efforts have historically focused on technology development, market penetration, and commercialisation. Limited work has been done to look specifically at the conditions and factors necessary for the integration of AI technologies into routine clinical care [14, 17]. While technical problems (e.g., performance, unreliability) have been regularly put forward as a reason for the difficulties of integrating these technologies into healthcare organisations and systems [26], they explain only a small part of the problem. Broader socio-technical conditions and factors rather explain many of these difficulties [18, 26].

The social scientific literature on health innovations has shown that the introduction of technologies into healthcare organisations and systems is a complex phenomenon [27]. This is particularly true for many AI technologies, which are sometimes described in the medical literature as disruptive innovation due to their evolving and autonomous nature [28–30]. Their implementation and use may require rethinking and/or redesigning existing governance frameworks and care models as well as new clinical, organisational, regulatory, and technological processes, business models, capabilities, and skills [18]. These changes involve, and impact on, a variety of stakeholders who may have divergent or even antagonistic expectations, goals, and visions towards technology [31–36].

To contribute to addressing current knowledge gaps, the goal of this study is to explore and understand the challenges of integrating AI technologies within a large academic hospital in Canada (referred to as “the City hospital”). We aim to answer two questions:

- How do multiple interacting influences facilitate and constrain the integration of AI technologies within the City hospital?
- What learning can we derive for policy and practice for better integration of AI technologies in healthcare organisations and systems?

The study is not limited to a specific AI technology or clinical area but encompasses all 87 AI technology-based initiatives developed and used to varying extent in this hospital. Where relevant, we specify the type of AI involved to contextualise the factors, conditions, or challenges described.

## Theoretical framework

**Fig. 1:**
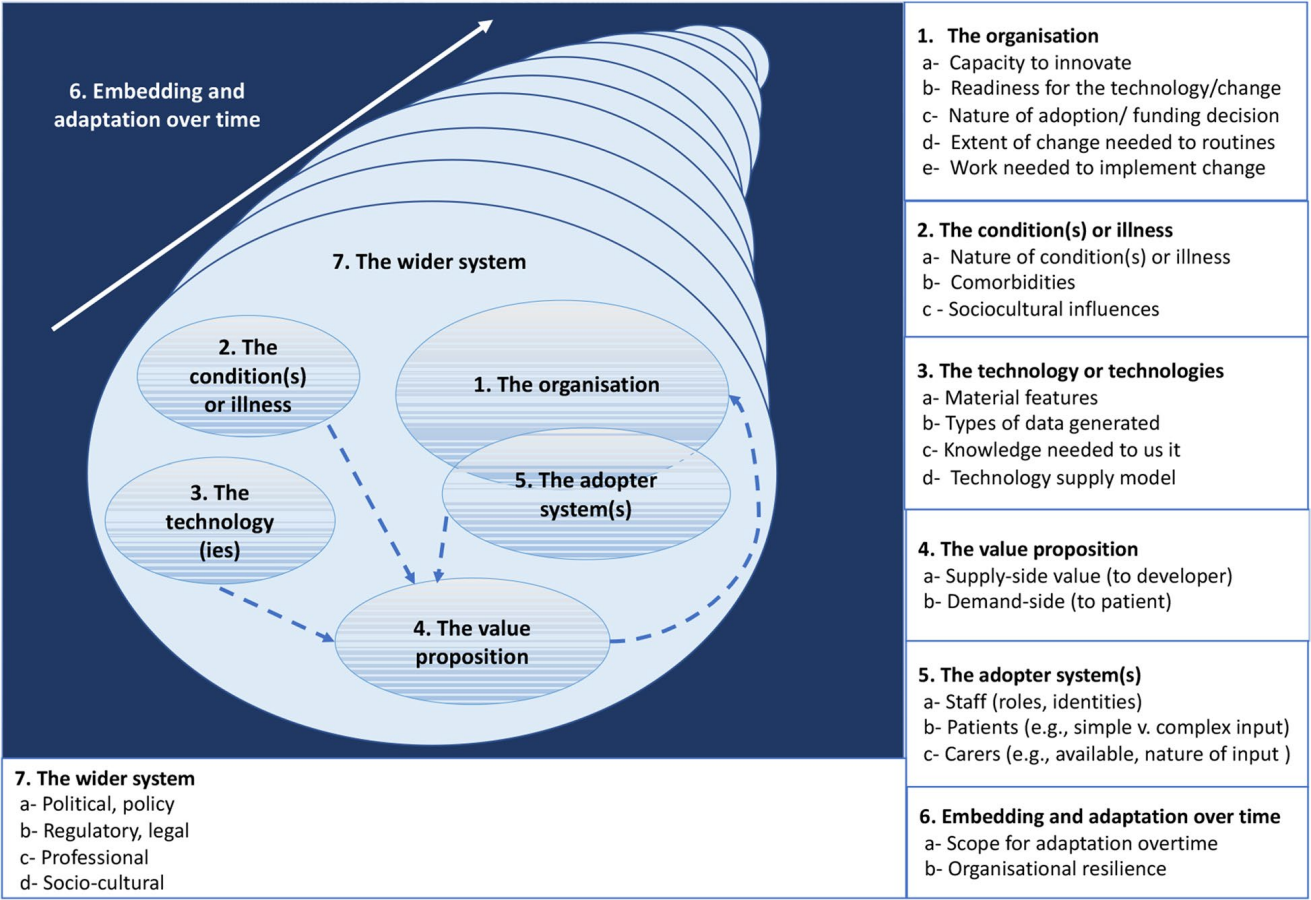
An adapted version of the NASSS framework (adapted from Greenhalgh et al. [27])

To make sense of the complexity underpinning the AI integration efforts in the City hospital, we used an adapted version of the Nonadoption, Abandonment, and challenges to Scale-up, Spread, and Sustainability (NASSS) framework developed by Greenhalgh et al. [27], which supports an exhaustive sociotechnical approach to health innovation. Following this adapted version, we present the seven dimensions of the framework in a different order from the original version in order to make sense of the narrative within the organisation studied, thereby covering: 1) the organisation; 2) the condition(s) or illness; 3) the technology or technologies; 4) the value proposition; 5) the adopter system(s) (e.g., staff, patient, caregivers); 6) embedding and adaptation over time; and 7) the wider system [27]. See Fig. 1 for a description of the seven dimensions.

There were many reasons for adopting the NASSS framework over other frameworks. First, it stems from a hermeneutic systematic review, supported by empirical case studies of technology implementation in healthcare [27, 37], and its key strength lies in its synthesis of 28 technology implementation frameworks, that is informed by several theoretical perspectives [27, 37]. Second, it was developed to fill an important gap “on technology implementation—specifically, to address not just adoption but also nonadoption and abandonment of technologies and the challenges associated with moving from a local demonstration project to one that is fully mainstreamed and part of business as usual locally (scale-up), transferable to new settings (spread), and maintained long term through adaptation to context over time (sustainability)” [27, 37]. Third, in contrast to the deterministic logic of many existing frameworks, the NASSS framework is characterised by its dynamic aspect, particularly in terms of interaction and adaptation over time. Indeed, a large part of the literature in the field has a tendency to “assume that the issues to be addressed [are] simple or complicated (hence knowable, predictable, and controllable) rather than complex (that is, inherently not knowable or predictable but dynamic and emergent)” [27, 37]. Therefore, major failures of large and ambitious technology projects may be underestimated and their complexity for healthcare organisations and systems tossed away [27, 37]. Fourth, whereas decision-makers and technology promoters as well as a part of the specialised literature often adopt a linear, predictable, and rational vision of change [38], the sociotechnical stance of the NASSS framework highlights the importance of examining how technology and the changes associated with it are perceived, interpreted, negotiated, and enacted by individuals and groups [33, 39, 40]. The same applies to AI technologies that may require transformation and/or redesign of services, a profound reconfiguration of clinical and organisational practices, and challenges to professional identities and practices [17, 33, 40]. Certain types of AI technologies also evolve autonomously over time – a particular characteristic that can be explicitly conceptualised through the NASSS framework [27, 41]. Overall, the NASSS framework was developed to be used reflectively, to stimulate conversation and generate ideas, which is one of our study’s aspirations.

## Methods

We conducted a qualitative study within the City hospital (Quebec, Canada) [42]. The latter had initiated several projects to integrate AI technologies in its care and service offer. Decision-makers and managers expressed a need for (independent) insights into the micro-, meso-, and macro-level systemic implications of the integration of these technologies within the organisation [43].

### Presentation of the organisation

The City hospital is one of the largest academic hospitals in Canada. It offers specialised and sub-specialised services to adult patients. It treats around 500,000 patients annually. It employs over 14,000 people. It also houses one of the largest medical research centres in the country, with an academic mission to produce and disseminate knowledge and research results. It also presents itself as an organisation with state-of-the-art facilities and equipment. It has been ranked by the U.S-based magazine Newsweek as one of the world’s top 250 Best Smart Hospitals for 2021. It hosts one of the largest annual digital innovation events in Canada.

At the time of the study, the City hospital had over 115 digital health projects (Table 1), with 87 of these involving AI. Around 95% (≈82/87) of the AI technologies were in the development/experimentation or early implementation phase. Only four were integrated into services. Approximately 72% (≈62/87) of the AI technologies identified within the organisation were for the diagnosis, treatment and/or monitoring of complex chronic or acute conditions: cancers, neurological (e.g., epilepsy), and ocular conditions.

**Table 1:** Overview of AI technologies within the studied healthcare organisation (March-July 2021). The figures are indicative. They may have changed since the time of data collection. (^a^Other technologies were reportedly discontinued or paused, but not referenced at the time of data collection)

**Technologies and purposes**	**Development and experimentation**	**Implanted or in implementation process**	**Sustained**	**Withdrawn**
**Decision-making or decision-making support: diagnosis/treatment/follow-up of patients**	48	10	4	-^a^
**Optimisation and organisation of human resources**	9	4	-	-^a^
**Administrative, material and/or logistical**	3	1	-	-^a^
**Training, learning and knowledge transfer**	3	2	-	1^a^
**Other purposes**	1	1	-	-^a^
**Digital projects, but without AI**	11	13	4	-^a^
**Total projects**	115 (including 87 AI projects)			

### Recruitment

We identified a purposive sample of key stakeholders, with the aim of capturing diverse perspectives and experiences [44]. We conducted internet searches and consulted reports and documents produced by the City hospital to identify potential participants, who were drawn from distinct roles and varied levels of involvement in the development, implementation, and use of AI technologies.

A personalised invitation email was sent to each potential participant explaining the project and why they were invited to participate. Two reminders were sent in case of non-response. Respondents were invited to indicate other participants (i.e., snowballing) [45]. This resulted in a sample of senior and middle managers/decision-makers, clinicians (e.g., physicians, nurses), clinicians/informaticians/researchers, technology assessment specialists, procurement specialists, lawyers, patients, and technology providers. Patients were identified through patient partners (volunteers) collaborating with the City hospital. Of the 42 invitations sent, 29 people agreed to participate. Table 2 shows participant profiles, many of whom cumulated multiple professional and/or experiential backgrounds.

**Table 2:** Summary of the study participants’ characteristics

**Type of participants (category of respondents)**	**N**
**Decision-makers/senior managers**	3
**Intermediate managers working on innovation and technology projects**	6
**Clinicians/managers working on innovation and technology projects**	2
**Clinicians/informaticians/researchers working on innovation and technology projects**	5
**Procurement and contracting specialists working on innovation and technology projects**	2
**Jurists and/or lawyers working on innovation and technology projects**	1
**Health technology assessment specialists**	1
**Patients**	4
**Technology providers**	5
**Total**	**29**

### Data collection

Between March and July 2021, the first author (HA) conducted 29 interviews in French (27) and English (2), using the Zoom™ videoconferencing platform (interviews lasted 30–90 min). Prior to the interview, a consent form summarising the objectives of the project was shared. Interviews were audio recorded with the permission of the participant and transcribed verbatim by HA. The questions were formulated according to the dimensions of the NASSS framework and informed by documents shared by the City hospital (e.g., list of projects and technologies). HA first tested the qualitative interview guide with two respondents prior to the start of the study. No major revision of the initial version of the guide was required. He took notes during and after the interviews and subsequently used them to contextualise the analyses. The interview guide slightly evolved depending on the participants’ responses as new information emerged. By adapting the interview guide, we were able to capture both expected and unanticipated tensions and practical challenges, grounding the discussion in participant experiences to avoid vague or abstract responses. Given that the same person (HA) co-developed the guide and conducted the interviews in French and English, this minimised the risk of variability that could arise from having different people collecting data in different languages. Interview data and document analysis, alongside our knowledge of the context (team members have been involved in various research and evaluation projects on digital technologies and innovations in Quebec and Canadian healthcare organisations and systems for several years) guided triangulation of data sources [46].

### Data analysis

Data were coded and analysed with Dedoose™ software. HA performed the first round of analysis and developed a preliminary coding scheme. In the second round, the scheme was refined, challenged, and discussed iteratively by the second author (PL) [43]. We conducted a deductive-inductive thematic analysis. The deductive analysis was guided by the NASSS framework (Fig. 1) [27]. Drawing on its seven dimensions, we created codes to capture the micro, meso and macro-level challenges and implications associated with the integration of AI technologies in the City hospital. The inductive analysis aimed to capture emerging themes not covered by the framework [44, 47]. After agreeing on the different themes identified, we concluded that none required the addition of new dimensions, as all identified themes fitted within the NASSS framework. Data saturation was reached for the themes and observations reported in the findings. Given the importance of context in the NASSS framework, we sought to understand and clarify the contextual elements where respondents had different views or judgements. We decided not to disclose certain details either because the participants requested it or to ensure confidentiality. However, this information was useful to contextualise and better understand other findings and events. Our findings are illustrated with participant quotes organised around key themes of the NASSS framework (translated from French to English when needed) (Table 5 in Appendix). The letter P used in quotes refers to “participant”, followed by numbers designating the order in which interviews were transcribed.

Findings are reported as a narrative account [48]. This is critical in allowing us to capture the complexity of the subject, the explanatory and interpretative dimensions, and the varied stories and perspectives gained from participants in making sense of the issues around the adoption of AI technologies.

### Results

We present the findings according to the seven dimensions of the adapted version of the NASSS framework (Fig. 1). To ensure fluidity in the presentation of the findings, the participant roles are used as a general category to help the reader identify certain tensions between the viewpoints and perspectives expressed. In this sense, there is no pretension of generalisation given the small number of respondents in each category. The analyses are intended primarily to provide high-level dynamics related to each dimension of the NASSS framework and not those specific to the types of AI discussed.

### The organisation

For the technology providers we interviewed, the City hospital has several internationally renowned clinicians, both in the clinical field and in the use of AI. Several managers and clinicians also reported that senior management is known to value and encourage technological innovation, which has led to the creation of a “data lake” that allows the integration of data from different clinical systems (e.g., clinical records, laboratories, vital signs, imaging), which is a major asset for the development and/or validation of certain AI technologies. According to technology providers, access to the specialised expertise of clinicians who know the data is as important as access to the data itself. These clinicians play an important role as a trusted guarantor (or legitimising authority) for AI with other clinicians, decision-makers/managers, patients, and citizens. In the words of one clinician-manager, the relationship and communication between these clinicians and the City hospital’s senior management is generally perceived as positive. He pointed out that this synergy helps to mitigate some of the issues and conflicting visions and expectations of AI.

According to a technology provider, because of the characteristics of Quebec’s single-payer and universal health system, the City hospital allows for holistic management of patients suffering from several pathologies or requiring different care and treatments. He added that this unique advantage enables the development of AI technologies with a broad spectrum of action (i.e., compared to those developed in contexts where care is fragmented between different hospitals and/or clinics). Despite this asset, there is a broad agreement among the interviewees that the City hospital is characterised by significant complexity that has the potential to impact its ability to realise the value promise of AI technologies.

Use of AI technologies in the City hospital necessarily involves different departments, committees, and stakeholders (e.g., Information technology -IT- department, procurement department, project office, professional services department). According to several managers, clinicians, and industry providers, the roles and mandates for these different groups and stakeholders are not always clear. Coordination and communication between teams and/or departments are sometimes difficult or non-existent. According to a manager, this results in confusion and tension about expectations, visions, and responsibilities. He pointed out that difficulties experienced by some AI projects were due to a department or committee not being engaged at the right time (e.g., as a result of legal and/or procurement framework, Cloud storage space, professional services department). For managers and clinicians, a horizontal body should have been established to coordinate and ensure coherence and communication between the different initiatives and stakeholders, with the aim enabling mutual effort, coordination, and accountability. For another manager, by ensuring an initial screening of technologies proposed by industry, such a body would avoid the influx of useless technologies to clinical teams and associated time and resource costs.

Both industry and organisation respondents agree that the City hospital doesn’t always have the capacity to meet the initial and recurring costs and investments required for the successful integration of AI. To overcome this funding problem, at least partly, an interviewee told us that the organisation is obliged to open its doors to industry for co-development, or as a testing ground, of AI technologies. This sub-contracting allows the City hospital to benefit from a free user licence for a fixed period or for life. However, it was reported that this partnership contracts model (e.g., co-development or serving as a testing ground for the industry) is likely to lock the organisation into a technology-centric logic, with no real margin of manoeuvre to choose technologies that really meet its needs. There are multiple projects under this partnership model within the organisation. Several technologies could simply end up being only partially developed because the technology provider has withdrawn, or the technology was abandoned. Within such a context, several managers and clinicians recognise that it is difficult to create a real organising vision that supports and enables AI within the City hospital.

According to managers and clinicians, these partnerships with industry imply an over-solicitation of the clinical teams as, in addition to their clinical and administrative work, they must dedicate time to testing and experimenting with the various technologies presented by the technology providers. In this regard, several organisation and industry respondents pointed out that clinicians in the City hospital are not valued or remunerated for their contribution to the development and/or experimentation of technologies. It is not uncommon for some clinicians to feel that industry benefits from their clinical expertise without any real return on investment for them. Technology providers interviewed refuted this point. For them, the difficulties in integrating their technologies into the organisation are essentially due to the opposition of some influential clinician-researchers who are themselves developing in-house similar technologies. In the words of one industry respondent, this is a conflict of interest and unfair competition. Nonetheless, technology providers support the importance of creating incentives to encourage clinicians to collaborate with industry. On their part, several clinicians and managers consider that the organisation should value in-house initiatives more highly because they emerge from the needs and expectations of the field. However, there is agreement that the organisation does not have the financial and human resources to support these initiatives. In addition, according to one manager, as a public entity, the City hospital does not have a mandate to develop and/or commercialise technologies. At some point, a company would have to be involved to ensure commercialisation.

Managers, clinicians, and industry acknowledge that the nature and extent of the changes associated with the integration of AI within the organisation are still largely unknown. For example, it is very difficult to assess financial implications over time. Two managers reported that the City hospital paid an additional CA$20,000 to CA$30,000/year for the storage and management of its data. This cost was not initially budgeted but subsequently required by the Cloud service provider who had estimated the size of the data. According to the same respondents, such “little surprises” could lead to some technologies being abandoned along the way, even if they are clinically relevant, either because the organisation cannot afford the costs or the Quebec’s Ministry of Health and Social Services (known as MSSS) refuses to cover them.

Both industry and organisation respondents reported that many AI technologies require access, sometimes in quasi-real time and without human intervention, to large amounts of data of various types. Unanimously, interviewees acknowledge that the organisation’s rules and procedures do not currently allow this (or very barely). Technology providers are calling for easier access to data. However, on the organisational side, several managers consider that such rules and procedures need to be further strengthened. Some of them emphasised the importance of having a Specialist digital lawyer to ensure that these issues are addressed when contracts are signed. They also add that there should also be a Chief data officer to ensure adequate and coherent governance between the various initiatives that involve clinical-administrative data.

### The condition(s) or illnesses

Most of the AI technologies identified (72% ≈62/87) within the City hospital are directed at the diagnosis, treatment and/or monitoring of complex chronic or acute conditions (e.g., cancers, neurological, ocular conditions) (Table 1). These conditions generally require ongoing or periodic support and monitoring over long periods of time with significant implications for patients and their families, and for the financial sustainability of the healthcare system. They also require complex, individualised, and evolving service models to continue to meet the needs of patients and their families. Several interviewees underscore that the use of AI could reduce waiting times and the costs of managing these pathologies. For a technology provider, these technologies are also expected to help identify new patterns and digital biomarkers that would facilitate the diagnosis and treatment of poorly characterised and/or unpredictable diseases.

For several respondents, this focus on specific diseases is partly due to the nature of the technologies available on the market. These technologies are addressing pathologies mainly through image analysis and/or signal quantification. This makes them more easily measurable, therefore more attractive to technology developers seeking rapid market access.

### The technology or technologies

There are diverging perceptions between clinicians, managers, technology providers, and patients on what makes AI attractive, reliable, and mature enough for clinical use and/or interoperable with existing systems.

According to a manager, some of the technologies proposed to the City hospital under the label “AI” are, in fact, expert systems with advanced calculation software. Branding the products in this way is a strategy used by some companies to attract investment and/or obtain contracts. While an AI designation increases the market value of the technology, it does not necessarily increase the clinical value. For another manager, this labelling of AI products is also partly due to the organisation’s pressure on technology providers to integrate AI. This is a significant step for technology companies as, compared to traditional software, AI technologies require specific regulatory requirements, technical infrastructure, expertise, and resources.

Several participants raised emerging security issues specific to AI. This is not only about the security of the technology and infrastructure, but also about the security of the algorithm itself. The latter could be hacked and modified, which can have a direct clinical impact on the patient. According to a manager, being able to recombine data from different sources, AI technologies could easily re-identify individuals. On their side, technology providers pointed out that these security issues are mainly due to the City hospital’s obsolete systems and technology infrastructure. They underscore how their technologies conform to the best security and quality standards and norms on the market, and that unlike public organisations they have the best IT expertise. An industry respondent added that, since the customer is the guarantor of their added value on the market, they also regard data security as central to their reputation and brand image. If an incident occurs, the company could simply lose customers or even go bankrupt.

Some AI technologies need to run on an integrated technological platform or operating system (e.g., electronic health record -EHR-) that allows for optimal data flow and exchange between the different technological systems and organisational departments as well as across healthcare system organisations. Respondents agree that the City hospital’s departments generally have outdated and disparate systems and infrastructures that are frequently not interoperable. However, several managers, clinicians, and technology providers argue that this is a common problem for the whole healthcare system, as an integrated and interoperable EHR does not exist. In this regard, for a population of over 8 million people in Quebec, there are over 30 million patient identification cards. A patient may have several cards with a fragmented EHR in several organisations. Similarly, one interviewee stressed that the equipment used (e.g., scanner, magnetic resonance imaging -MRI) in the City hospital does not always meet the requirements for AI. In some situations, it is difficult to know where the data is, or how it is processed and collected by certain technologies or equipment. Problems with internet connection and data transmission via Wi-Fi are also reported.

There is a consensus that AI technologies need high-quality data. Both industry and organisation respondents highlighted that a significant amount of clinical-administrative data (e.g., handwritten clinical notes) and patient records are still scanned in portable document format (PDF), which is not usable for planned AI. For technology providers, the meaningful use of data, which raises the question of the purpose of the data collection, is missing within the organisation and should be given more consideration.

For its AI programme, the City hospital works with many specialised start-ups and small- and medium-sized enterprises (SU/SMEs). One such technology provider stresses that the survival of their company depends on their ability to seek liquidity in the financial market (e.g., venture capital). This means that they are necessarily accountable to their shareholders who may be looking for the fastest and most profitable exit events possible (i.e., when an investor sells his/her shares in a company to collect cash profits). This approach brings challenges for the City hospital in terms of working relationships, technology development, and continuity of care. For instance, SU/SMEs can be bought by multinationals or simply disappear (e.g., bankruptcy), or a company may stop a technology or cease to update it. According to a manager, the City hospital does not necessarily have the capacity to maintain these technologies on an ad hoc basis or replace them with others. Another interviewee added that sometimes the organisation has no guarantee of recovering data hosted or operated by these technology providers or their subcontractors (e.g., Cloud services).

### The value proposition

Stakeholders interviewed have divergent definitions of what constitutes the perceived, anticipated and/or actual value of a technology and the parameters to be considered for measuring it (e.g., safety, efficacy, and effectiveness criteria). About 95% were still in development/experimentation or implementation.

Several technology providers mainly express the value of their technology in terms of its potential to improve healthcare and its efficiency. They pointed to significant consumption of resources by the healthcare system while at the same time being unable to meet the healthcare needs of the population. For these interviewees, AI can solve the problem whilst modernising the healthcare system. In this regard, for a supplier, to realise such value, the City hospital, and the healthcare system in general, must be willing to take some risks. He stressed that if the latter wait for AI to be perfect and risk-free before using it, the technology will never be integrated, and its value promise never delivered to the population.

A manager reported that many AI technologies in the City hospital were at a value promise stage (i.e., with anticipated, rather than actual value stage). Other interviewees consider that this value promise remains relatively speculative, based on vague projections and estimates. In this regard, from the organisation’s perspective, the perceived value of AI technologies is mainly about improved clinical quality and safety, and performance. The expectation to achieve this value is to have tailor-made AI technologies adapted to the setting, clinical contexts, and ways of working. However, focusing on tailored AI solutions can sometimes be a major constraint for technology providers. According to several interviewees, suppliers prefer to commercialise generic technologies that can be easily marketed elsewhere with minimum modification (plug-and-play). Several managers and clinicians added that the costs involved in implementing and adapting the technology to the local context are regularly underestimated by these suppliers. The latter often lack an understanding of the complexity of clinical practices. For example, one company stopped working with the City hospital because it considered that its clinical needs are too specific for the AI technology to be cost-effective.

Because of its status as a leading academic hospital, the City hospital is highly sought after by the AI industry. Several interviewees recognise that the organisation is used to showcase and legitimise the technology’s value proposition, hence its market value and potential for widespread commercialisation. A technology provider also reported that the organisation serves as a gateway to the healthcare systems of Quebec and other Canadian provinces. At the same time, according to organisation respondents, the City hospital benefits from media coverage, which gives it a competitive advantage in attracting talent and expertise. However, divergence over the actual added value of certain technologies may constitute a source of tension between senior management and clinical teams. Some AI technologies are likely to exacerbate workload and staff burnout (e.g., technologies intended for the optimisation of clinical-administrative processes). For a manager, since AI technologies are still considered over and above other priorities, their impact on the quality of work and clinicians’ satisfaction is not really taken into consideration in the organisation’s assessment of their value (e.g., flexibility, alignment with clinical-administrative workflows). He added that the City hospital has difficulty in moving the value of these innovations from the Triple Aim to the Quadruple Aim: “improving the patient experience, the population health and the quality of work and satisfaction of healthcare providers, and reducing costs” [49].

The organisation’s clinical-administrative data, which is used to develop and/or operate some AI technologies, may contain biases and may not be representative of the general population. For several interviewees, AI technologies may also not respond to the contextual realities and needs of some populations (e.g., indigenous, rural, or minority people). Patients and organisation respondents also pointed out that these populations are rarely involved in the design, development, and implementation of AI technologies within the City hospital. Several interviewees recognise that assessing the added value of AI technologies by population segment is essential, but very difficult to achieve.

#### The adopter system(s)

Interviewees overwhelmingly agree that certain AI technologies could have a direct impact on the patient-clinician relationship. Some progressive diseases require human care and support over time. For AI technologies designed to monitor chronic diseases, some patients fear being lost from sight by their healthcare providers. According to several patients, it is important to ensure that they always have the possibility of in-person meeting with their clinician. As a patient pointed out, technology could never understand their subjective experience with the disease better than the clinician. For this and another patient, listening and empathy are sometimes more important in a care pathway than medication and technology. They mentioned that the therapeutic relationship goes beyond the simple dimension of the disease.

According to a patient, some patients registered with the City hospital can have up to 5 technology applications, sometimes non-interoperable. Some of these technologies do not operate on older Apple- or Android-supported smartphones, making it hard for several patients to use them unless they upgrade their hardware. Technologies may also require access to patient-generated data at home. Patients, clinicians, and managers stressed that patients may not have the technology and equipment and/or a good internet connection, but also the social and cultural capital (e.g., literacy, family network) to fully benefit from the potential of these technologies. They recognise that these technologies could lead to additional costs and expenses for these patients. Even when they have the technology, they may need technical support at any time of the day (24/7) as the disease “has no working days”, as a patient notes. This support is not automatically provided by the organisation and not all patients have a family/friend network that can be mobilised when needed. Paradoxically, technology could exacerbate the disease burden for these patients.

Several respondents reported that the adoption and use of certain AI technologies typically requires a reorganisation, or even a redesign, of clinical practices, of the organisation of services, and of the modes of governance and control within the City hospital. According to clinicians and managers, these changes could be associated with a feeling of loss of professional autonomy, identities, values, and skills. In the words of a manager, AI technologies could cause an erosion of information asymmetry (in favour of the organisation and the MSSS) and challenge clinicians’ autonomy of practice. The erosion and reduction of the scope of expertise due to the replacement of part of the clinical activity by AI was also pointed. However, several respondents relativised these fears, stressing that it is rather the clinicians trained in AI (e.g., clinician-informatician, clinician-data scientist) who will replace the others. This new expertise will have to be institutionalised and valued. This could imply a revision of the boundaries of professional jurisdictions (e.g., reserved acts) and of certain negotiated orders and privileges, and therefore of powers (e.g., nurse vs. general practitioner; general practitioner vs. specialist physician). Managers and technology providers pointed out that a technology that provides real added value for patients will never be integrated into practice if clinicians perceive it as a threat to them.

It was reported that the effort to integrate AI within the City hospital is occurring in a context where clinicians are under great pressure with high workloads. Some emphasised that they have no time to waste on these technologies, particularly those imposed on them by senior management and/or industry. They also expressed a feeling of innovation fatigue. Managers and clinicians acknowledge that this lack of time, but also of engagement, has a negative impact on the success of technology training and promotion initiatives within the organisation, and therefore its subsequent adoption and use. In addition, clinicians involved in technology integration efforts are mainly volunteers (e.g., champions, super-users). As the contribution to innovation is not considered a clinical activity, it is not remunerated nor recognised in their performance indicators. According to several clinicians and managers, this point is a significant barrier to clinicians’ engagement, especially to embrace the necessary changes and adaptations, and to construct meaning and develop new identities with regards to AI.

There is agreement that the need for continuous monitoring and follow-up of some AI technologies in everyday clinical practice made the role of IT teams more critical to clinical practice. According to a manager, this is a major change as clinical and IT teams have historically evolved in silos. In this regard, it is difficult to align cultures and languages within the City hospital in the midst of developing AI technologies and services. For some clinicians, the increasing adoption of AI in their practice may make them dependent on IT teams (potentially conflicting with their autonomy of practice). To address this issue, an interviewee emphasised the importance of the presence of translators or boundary spanners with a hybrid clinical-IT profile to bridge and build a healthy collaborative space between clinical and IT teams. These translators could also act as a bridge between clinical teams and technology providers. The same respondent reported that such a role is already played by members of the City hospital’s Innovation Pole team and several clinicians.

Several managers and clinicians, acknowledge that the blind confidence and lack of critical distance could affect the use of certain AI technologies in clinical decision-making. In this regard, they see the problem of transparency and explainability of AI decisions (black box). According to an interviewee, the problems of data quality and bias are serious enough to be doubly vigilant on this point. A technology provider recognised the importance of clinicians being able to understand how the decision is made by the AI (e.g., parameters retained or excluded) and whether such a decision is right or wrong. To do so, clinicians may need technical support from AI experts, which the City hospital does not necessarily have. According to several respondents, it is difficult for public organisations to recruit AI experts, as the latter are more attracted by the private sector where working conditions and remuneration are very advantageous.

#### Embedding and adaptation over time

The City hospital’s IT systems are theoretically well secured for AI or associated technologies needed for its functioning. Indeed, any new technology for clinical-administrative use should meet strict criteria for safety and effectiveness. They should be licensed and/or authorised by the IT department or regulatory agencies. However, several managers and clinicians recognise that, once implemented, numerous technologies are not necessarily monitored and controlled over time. The result is a complex, fragmented, and non-interoperable technology environment that is difficult to manage and update, but also vulnerable to cyber-attacks. Some AI technologies are likely to dysfunction and/or operate and evolve awkwardly in such an environment, which could pose patient safety issues.

According to industry, clinicians, and managers, the lifecycle of AI technologies (i.e., the period during which they can function adequately without major upgrades and avoid replacement by new and better technologies) is often very short, and potentially only a few months. The City hospital should be able to upgrade its technology systems and equipment continuously. The costs can be significant. In this regard, equipment and devices (e.g., scanner, MRI) required for the functioning of certain AIs may be considered obsolete after only five years of use. The data they generate is no longer usable, which has a direct impact on their clinical reliability (e.g., ability to detect cancer). To remedy this problem, some technology providers offer to lease equipment. According to the latter, City hospital could then benefit from the latest equipment, with embedded AI, with no obligation to purchase. A technology provider explained that such a model involves the organisation to engage in service contracts over varying periods of time with the supplier. Such contracts usually include the implementation, maintenance, and upgrading of the equipment and associated technologies. The same respondent emphasised that this proximity model would also allow for a feedback process, necessary to adapt to the evolving needs and expectations of clinical teams. However, for several managers, this model raises concerns about the risk of locking the City hospital into a dependency relationship with a single supplier. They reported that this “chaining” could, among other things, increase the supplier’s control of the organisation’s data. To illustrate this point, an interviewee indicated that a technology provider has already “forced” the City hospital to pay for access to its own data (hosted/stored on the supplier’s servers). The same person reported that suppliers want to benefit from an annuity/rent, i.e., a continuous flow of money over time.

#### The wider system

A gap exists between those who call for a pragmatic approach (e.g., test-and-error, sandbox logic) and those who call for the consolidation of the precautionary principle (i.e., decision-makers adopt precautionary measures when scientific evidence about a human health or environmental hazard is uncertain and the stakes high) [50]. For several suppliers, the precautionary principle is a major obstacle to the integration of these technologies into the healthcare system. They stressed that regulation should be made more flexible, because zero risk does not exist in healthcare. An interviewee pointed out that the autonomous and evolving nature of some AI technologies will inevitably lead to failures and unforeseen incidents. Instead, lessons should be learned from these malfunctions and incidents to improve the technology. The Post-Market Approval/Post Market Surveillance model adopted in the USA was given as an example. This approach is rejected by other several managers and clinicians who consider that the lives and safety of patients cannot be subject to “hazardous test-and-error”.

Respondents are unanimous in stating that the authorisation, contracting, and financing process of AI technologies by the MSSS, which mainly focuses on the initial purchase price (capital equipment, which results in the procurement of technology with a fixed price, often the lowest, of which the organisation becomes the owner), is no longer adapted to the reality of AI technologies (Table 3). Firstly, many AIs operate with a “Software as a Service (SaaS)” business model. It is a monthly or yearly subscription for the organisation. According to technology providers, this model is justified by the fact that these technologies require continuous monitoring, control, and maintenance over time. Some respondents also called for the adoption of the “Value-Based Procurement (VBP)” business model. In this case, the suppliers are paid according to the value generated by their technology (e.g., 10% of the savings made over a patient’s entire care and service cycle). As these technologies are not cheap, there is a risk that they could be excluded from current tendering processes. According to several managers, the tender model does not consider the costs required for the implementation and adaptation of the technology to the local context. Examples where additional costs were required at the time of implementation, not initially foreseen, are relatively common. However, interviewees recognise that VBP is still difficult to implement. Because of the evolving nature of certain AIs, their value could change over time. Currently, it is difficult to ensure their continuous evaluation and monitoring due to the fragmentation of services and the lack of an integrated EHR, as well as trained and qualified human resources (e.g., collection, organisation, structuring, visualisation, and analysis of AI technology usage data), among other things.

**Table 3:** Description of the two acts governing the public procurement of technologies in Quebec’s health system

In Quebec, digital innovation is mainly regulated by two Acts:
1. The Act on IT systems and infrastructures, which is specific to the acquisition of digital technologies by public organisations. Within the framework of this Act, when a public healthcare organisation intends to spend part of its operating budget to acquire a technological system, it must justify the need to the MSSS. The MSSS’s IT division (known as DGTI) must validate an opportunity file (4 pages: What do you want to do? How are you going to do it? How much does it cost?). It takes approximately 5 months for the DGTI to give its initial approval. Next, the organisation must submit a business case (15 pages) with an in-depth analysis. This second step takes around 8 months. During this process, the organisation does not receive any funding and cannot do anything. For both organisation and industry respondents, this is potentially a significant deterrent to innovation and slows adoption and use of innovations. According to an interviewee, such a process should not take more than 3 months to be in line with the reality of AI technologies
2. The Act on contracting by public bodies (known as LCOP), which regulates the procurement of equipment or technologies by public organisations. It provides a framework for service contracts between public healthcare organisations and equipment and/or technology providers. For contracts that exceed CA$25,000, organisations must go through a public tendering process to select the lowest cost technology provider

According to several managers, the difficulty of acquiring certain AI technologies through the tendering process is another reason why the City hospital prioritises partnership contracts (e.g., co-development or serving as a testing ground) over service contracts (e.g., procurement of technology and/or associated services) with suppliers. In the words of a manager, as long as the organisation does not incur expenses (e.g., having the technology at no cost for a given period or forever) from its operating budgets, it does not have to justify its actions to the MSSS. This strategy also allows the City hospital to accelerate the integration of these technologies into its care and service offer by avoiding the complex bureaucratic process of the MSSS. However, some interviewees reported that partnership contracts do not always allow for the sustainable use of the technology beyond the free-of-charge period. In some situations, the organisation would have to incur expenses after this period and sign a service contract. It would then have to go through the tendering process again. If the latter is won by a different supplier, the initial technology should then be withdrawn, which condemns the City hospital to a kind of eternal restart.

Several technology providers argue that the tendering model is a barrier to entry into healthcare for SU/SMEs, although they could offer AI technologies with real added value. Unlike large companies, SU/SMEs do not have sufficient financial and marketing capacity to offer low prices.

Several respondents, both in the City hospital and industry, pointed out that the Act on the protection of personal information is also seen as a major obstacle to AI in the healthcare system. Typically, when a patient is treated in a public healthcare organisation, his/her consent does not include the secondary use of his/her data for research or other purposes. Legally, AI technologies developed or tested with this data cannot be used and/or commercialised, at least theoretically. According to an organisation interviewee, overcoming this barrier would entail considering that once a patient is treated in a public healthcare organisation, he/she automatically consents to the secondary use of his/her data for service improvement and research purposes. Several patients interviewed agree with this approach. However, they insisted that patients should always be able to withdraw their consent if they so want (opt-out).

Also concerning data, several interviewees highlighted the central role and necessity of Cloud services (e.g., data storage, exchange, and management) for optimal and effective use of AI technologies. According to a manager, Cloud services providers are mainly multi/transnational companies. The latter have servers and relay points all over the world, which means that data could travel across national borders. This challenges regulatory sovereignty. The same interviewee reported that Quebec legislation requires that data be hosted on servers located on its territory. However, the City hospital does not always have the levers to verify and ensure that the providers really respect this requirement. Nor does it always have the possibility of knowing whether an incident (e.g., security breach, data leakage) has occurred if the company does not communicate the information to it. In the words of another manager, “[The City hospital] does not always have the capacity to [ensure the security and reliability of the technologies], so it is forced to trust [the suppliers]”. In the same vein, it does not always have the levers and means to ensure that the technology provider has destroyed and/or deleted the dataset when requested to do so. In addition, according to another interviewee, the definition of responsibilities in the event of a patient harm incident is a not fully resolved issue yet. The latter highlighted that compensation could involve large sums of money that neither the supplier nor the City hospital would want to pay. In this regard, by simply being identified as a potential liable party in the event of an incident, the organisation or company could see the amount of its insurance contract increase considerably because of the risks involved.

Many AI technologies used in clinical decision making are considered as “Software as a Medical Device (SaMD)”. There is still no clear framework for their assessment and approval in Quebec and Canada. In addition, professional federations and colleges, and medical insurance bodies have not yet taken clear positions on their use in clinical practice. According to several interviewees, the absence of solid clinical practice guidelines, protocols, remuneration models, and professional responsibility frameworks limits the possibility of clinicians using these technologies. As an illustration, a manager pointed to the complexity of identifying responsibilities in the event of an AI error (e.g., misdiagnosis or mistreatment). Since certain technologies can decide autonomously, part of the responsibility of the clinician is transferred to them. For the same interviewee, numerous questions have yet to be answered: to what extent does the technology replace the clinician (totally or partially) or not? With the “black box” problem, AI does not always allow for tracing and understanding the decision-making process. Even when it is possible, technology providers might refuse to give access to their algorithm for commercial confidentiality and market competitiveness reasons. It is then difficult to know the nature and/or origin of the fault. Moreover, there is also the question of whether AI should imply an obligation of results, instead of the obligation of means to which clinicians are presently committed. According to another manager, technology providers prefer to classify their technologies outside the SaMD category. In this way, the clinician remains solely responsible in the event of harm. Then, the supplier avoids paying damages that may be substantial. Indeed, compared to a clinician’s error, which is usually limited to a single patient, an AI technology’s error could affect many patients. However, providers explained this choice by the fact that technology approval processes, such as SaMD, are time-consuming and very expensive.

Other regulatory constraints are pointed out by several interviewees. AI technologies never arrive ready for clinical use (plug-and-play). There is often adaptation and alignment work to be done. Some changes and/or adaptations are made informally (e.g., bricolage, workarounds) by clinicians. According to a clinician and a manager, these modifications are sometimes crucial in their decisions to use the technology or not. However, from a regulatory perspective, once licensed and authorised, a technology should not generally be modified, at least theoretically. Currently, any changes require the approval of the City hospital’s IT teams or of a governmental regulatory agency. Although justified in terms of financial and safety risks, there is a consensus among interviewees that this process is rigid, time consuming, and inadequate for the reality of AI. In this regard, updates to AI technologies should be quasi-automatic and continuous, in the spirit of how the iPhone works, often without human intervention. In the words of a clinician, any delay or blockage could have a direct impact on the diagnosis or treatment of patients.

According to a manager, aspects related to the organisations’ performance criteria and, therefore of their funding by the government are not yet fully defined for AI. In Quebec, the activity-based funding model is being deployed to complement the dominant historical budget model. This new model generally considers the activity of physicians (e.g., diagnosis, treatment, surgery), paid essentially on a fee-for-service basis, in the calculation of the budget the organisation will receive from the MSSS. The activity of other healthcare professionals, mainly salaried by the organisation (e.g., nurses), is not considered the same way (or only slightly) in these calculations. Numerous AI technologies intended for (or assisting in) diagnosis or treatment could be supervised by healthcare professionals other than physicians. The impact of this development on the funding of healthcare organisations remains unknown. In the same vein, the respondent highlighted the problem of the fragmentation of funding between medical, medico-social, and social services in Quebec. For example, some AI technologies have a clinical added value and are therefore covered by the MSSS. However, the latter does not cover other aspects such as the improvement of the patient’s quality of life (e.g., Quality-adjusted life year -QALY-). As a result, the City hospital could be required to solicit different departments, ministries and/or agencies to capture the different value components of the same AI technology.

According to several interviewees, funding from the federal government would have a direct impact on the integration of AI technologies into the City hospital. They report that federal programmes make it possible to fund expensive infrastructure projects, from several hundred thousand to several million CA$. However, implementation and sustainability are mainly under the responsibility of the Quebec MSSS because health falls under provincial authority in Canada. There is sometimes a gap between federal funding and provincial priorities. According to a manager, the Quebec MSSS does not automatically fund the implementation and sustainability of federally funded technologies. As a result, several technologies could eventually be abandoned. For another interviewee, one of the important limitations is that federal funding is often very targeted and specific to particular technologies and/or clinical areas. It does not provide sufficient flexibility for organisations to use it according to local needs and contingencies.

Lastly, several respondents recognise that inter-organisational collaboration for sharing expertise and experience is essential for AI. However, the fragmentation, lack of communication and coordination across public healthcare organisations make it difficult to establish such a collaborative environment. For example, according to a clinician, to develop AI technologies with real added value, it would be necessary to have access to large amounts of patient data. She explained that the way to do this, while competing with other technologies from other countries, is to pool the databases of different healthcare organisations in Quebec and Canada. Such an inter-organisational network is essential in the evaluation and approval process of AI technologies, as they are to be tested on data from different healthcare organisations (e.g., urban and rural hospitals, primary care clinics). For the same respondent, such multicentre testing would ensure reliability and effectiveness in different clinical and technological settings across the country.

## Discussion

### Summary of key lessons

Our study aimed to generate a better understanding of the conditions that facilitate or constrain the integration of AI technologies in a large healthcare organisation in Canada. By analysing a rich corpus of data using the NASSS framework, the study highlights seven lessons:

Firstly, an organisational culture and leadership that creates favourable conditions for AI is essential as well as the presence of clinical champions who act as ambassadors for AI. This is a lever to attract clinical and/or technical talent and expertise, but also companies in the field. The strategic alignment of the organisation’s clinical-administrative processes and infrastructures with AI technologies remains a major challenge. A lack of alignment could lead to partial integration of technologies or their abandonment, resulting in innovation fatigue among clinical and administrative teams. In a context where clinicians are over-solicited, they should be given the time needed to integrate the change, but also develop the professional expertise and identities that AI could require. It is also important that the technologies proposed to them are supported by evidence of improvements in patient care and services as well as in their work conditions and quality. The integration of AI within a hospital also involves a multitude of stakeholders whose activities and actions should be coherent and synergistic. Communication is fundamental to clarify roles, responsibilities, and mandates and requires a horizontal structure capable of coordinating actions and shaping a consistent organisational story about AI. The technologies proposed by the industry should be filtered so that those that really meet the needs on the ground are prioritised.

Secondly, financial and other incentives are needed to encourage clinicians to experiment and adapt these technologies to their practices. Investments in the development of AI technologies have so far focused on specific complex pathologies that present a great burden to patients and their families as well as to the healthcare system. To address these pathologies, AI mainly exploits image analysis and/or signal quantification, which makes it easier for suppliers to develop technologies and introduce them more quickly to the market. Yet, the sensitivity of safety and data protection issues implies that the hospital hires a lawyer specialising in digital technologies (to ensure that contracts are properly made) and a Chief data officer (for adequate and consistent data governance). Upgrading IT systems and infrastructure and recruiting new expertise hence require planning for both initial and recurring investments and expenditures.

Thirdly, the interoperability of AI technologies and the organisation’s systems and infrastructure are major obstacles to their routine use. Some technologies need quasi-real time access to data, which requires an integrated platform to ensure optimal data circulation between different IT systems and departments of the organisation, or even other organisations involved in the patient’s treatment. The qualification of some advanced software as AI could have financial and legal implications for the organisation. In addition to traditional clinical safety issues, the AI algorithm itself could be hacked and modified, resulting in harm to patients. By recombining data from various sources, individuals could be easily re-identified. These technologies could also require high-tech equipment with very short lifecycles, which the organisation may not have. Furthermore, many AI technologies are driven by SU/SMEs that could disappear from the market at any time. Hence, organisations should have the capacity to maintain the technology on an ad hoc basis or find an alternative and be able to recover and/or ensure the deletion of data by the initial supplier.

Fourth, the definition of the value of AI technologies is far from consensual as well as the expectations regarding what they can or should do. The ability to measure this value is of considerable complexity given the great contrast between the value proposition stated by suppliers, and sometimes by managers, and the actual value to clinicians and patients. The value of AI is not self-evident. Indeed, even if it has shown great performance in a laboratory context, this may not materialise in the real-world context of care and services. The value of some AI technologies also contrasts with the risks they raise given their evolutionary and autonomous nature. There are trade-offs between the precautionary principle, the need for some risk tolerance, and its clinical potential. Moreover, clinical practice may require very specific AI technologies, whereas suppliers tend to prioritise plug-and-play technologies with a potential for widespread commercialisation. The global value of AI could vary widely depending on the balance of the changes and transformations it requires and what it actually provides. This value may also change over time. Evaluating and monitoring AI’s value on an ongoing basis requires resources and expertise the organisation may lack, especially in view of the (re)production of bias across sub-groups of the population.

Fifth, contrary to the rhetoric about their potential to humanise care, some AI technologies raise concerns about the patient-clinician relationship and, therefore, about quality of care. The risk of mechanisation of care and the difficulty of physically accessing healthcare providers is palpable. Digital literacy, technical support, and change management for clinicians and patients using these technologies are essential. For clinicians, AI technologies may imply redesigning clinical practice and service organisation, but also new governance and control strategies within the organisation. Although improbable, there is a real concern that AI could partially or totally replace the activity of clinicians. Hyper-dependence on technology raises concerns about the erosion of clinicians’ expertise and the risk of blind trust in the decisions made by AI. As a result, clinicians may worry about being subordinated to the IT teams that would play a central role in the production of care. This new reality highlights the central role of translators or boundary spanners in building bridges and trust between clinical and IT teams, but also with industry. On a larger scale, the technology-driven approach to AI could cause a deterioration in clinicians’ work conditions and quality.

Sixth, the evolving and self-learning nature of some AI technologies makes time critical, distinguishing them from previous licensed technologies that do not generally require a new approval review. IT teams should approve and validate any changes or adaptations, and this becomes difficult with some AI technologies that evolve autonomously and update themselves. Any delay or blockage could threaten the diagnostic or treatment quality of patients. Continuous monitoring and control over time is required to avoid malfunctions and incidents, but also to make the necessary improvements. In this regard, the increasingly short lifecycle of software and hardware challenges the technical and financial capacity of the organisation to adapt and evolve its systems, equipment, and infrastructure at the right pace. Evolutionary AI technologies create the need for close and sustainable relationships between the organisation and the technology providers, a new relationship that: 1) requires solid frameworks to identify and resolve conflicts of interest as they arise over time; and 2) must avoid lock-in and dependence upon a single provider.

Seventh, many socio-political, economic, and regulatory factors are decisive in the integration of AI technologies, which are mainly offered under SaaS and/or VBP business models. These models are in opposition to the current tender model in Quebec that emphasises the cheapest technology (capital equipment). The legal framework of the current model constitutes a barrier to entry for SU/SMEs, some with high value-added technologies. Established bureaucratic acquisition processes are inadequate for the very short lifecycle of AI technologies. Consent requirements for the use of patient data are misaligned with this new reality and are prompting consideration of an opt-out consent model. AI technologies increasing rely on Cloud services mainly offered by multinational companies with servers and relay points all over the world. Data governance is even more important as healthcare organisations and systems have limited resources and tools to ensure that data management and storage comply with applicable laws. Identifying liability in the event of harm could therefore be very complex. AI technologies classified as SaMD, on the other hand, have specific requirements for quality, efficiency, and clinical reliability. To date, the lack of reference technologies makes it difficult for regulatory agencies to assess and approve them. Established mechanisms and processes are not adapted to the complexity and very short lifecycle of AI. Ongoing evaluation and monitoring mechanisms in the real-world context seem necessary, but the high degree of uncertainty associated with them requires a balance between the precautionary principle and a *laissez-faire* integration in clinical routine. Beyond the lack of clear frameworks and directives from the MSSS and other regulatory bodies regarding the use of these technologies by clinicians, inter-organisational networks facilitating the sharing of expertise and experience are essential. The current context is characterised by fragmentation, and poor communication and coordination between organisations and government agencies, which hinders an integrated and coherent vision of AI at the healthcare system: provincial- and federal-level of governance.

### Contribution to the existing literature

The results of this study contribute to knowledge in several ways. They shed a new and different light on the trend of recent years where the literature has mainly focused on the technical and promissory dimensions of AI. Our findings are consistent with those of Pumplun et al. (2021) and Petersson et al. (2022) who analysed implementation issues raised by AI technologies in healthcare in Germany and Sweden, respectively [3, 51]. Studies on telehealth and EHR also reported results that corroborate ours on AI [26, 31, 32, 34, 52–58]. In this regard, several authors pointed out the major contrast between the techno-optimistic discourse on the performance and efficiency of technology and the reality of services that are difficult to transform [56–58]. These experiences have shown that the difficulties encountered in the deployment of digital technologies are mainly due to the historical lack of attention paid to the sociotechnical factors and conditions necessary for their integration into healthcare organisations and systems. Hence, our study adds to the growing literature that considers technology in a complex sociotechnical transformation perspective that requires not only technological but also human, clinical, professional, organisational, socio-political, economic, regulatory, legal, and cultural changes [27, 40, 41, 56, 59–61]. Very limited attention has been paid to this perspective in examining AI to date, whereas our study clarifies its contribution and indicates some avenues for future research (Table 4) [3, 18, 26, 51].

**Table 4:** Some future research avenues

1. Definition and measurement of the value of an AI technology in a pluralistic context where expectations and objectives might be divergent or even antagonistic
2. Nature and impact of new models of care and service delivery for AI;
3. Design and assessment of appropriate models of inter-professional collaboration with AI;
4. Needs for new types and combinations of clinical and technical expertise and training for AI;
5. Nature and implications of business models in view of shifting remuneration and funding models for AI;
6. Design and robustness of data governance models (e.g., consent, storage, cybersecurity);
7. Management and sharing of intellectual property of the discoveries made by AI technologies exploiting healthcare organisations and systems’ data;
8. Definition of the legal status and liability of autonomous AI technologies in view of clinicians’ professional liability and obligations;
9. Mid- and long-term impacts on healthcare organisations and systems of venture capitalists’ influence over the lifecycle of AI-based SU/SMEs

From a theoretical standpoint, our study provides an original contribution to the literature on health innovations. It is one of the first to demonstrate that the NASSS framework is relevant for the analysis of the integration of AI technologies in healthcare organisations and systems [51]. The study contributes to the knowledge on the importance of a sociotechnical perspective to understand the complexity and unpredictability of transformations related to disruptive innovations such as AI [27, 51, 62].

### Implication for practice and policy

Our study provides new insights for decision-making and practice on the conditions required but also on the pitfalls to be avoided to ensure successful integration of AI technologies into healthcare organisations and systems. It shows that the pitfalls of the technocentric vision of digital health of the last thirty years in Quebec (and elsewhere too) could easily be repeated with AI technologies, but this time with more profound repercussions [31–33, 35, 36, 63]. As Matheny et al. (2020) highlighted: “Disconnects between reality and expectations have led to prior precipitous declines in use of the technology, termed AI winters, and another such event is possible, especially in health care” [64]. In this regard, the various stakeholders must be aware that AI is more an object of transformation at all levels of healthcare system governance, than a simple “intrinsically good/bad” tool. Its successful integration depends on several structural conditions, namely, appropriate: regulatory and governance frameworks; funding, business, and remuneration models; definition of the value proposition; management of conflicts of interest; governance of data; cybersecurity strategies; training and expertise, models of care and service delivery; inter-professional collaboration; and up-to-date infrastructure and equipment.

Specifically, AI highlights the importance of rethinking the collaboration between healthcare organisations and systems, on the one hand, and technology providers, on the other hand. Indeed, their interests sometimes represent competing financial and political objectives between which a difficult balance must be established [65]. Given their disruptive nature at all levels of the healthcare system, IA technologies could generate tensions and require trade-offs between perceptions, expectations, interests, and agendas that may be divergent or even antagonistic (ex. industry and venture capital, decision-makers, managers, clinicians, patients). These dynamics and power relations influence the trajectory of AI technologies in healthcare, either positively or negatively [59, 66]. Thus, if healthcare organisations and systems are not sufficiently equipped and prepared, “the AI landscape risks being shaped by early established companies and decisions made with insufficient evaluations in place due to pressures to embrace technology” [67].

In addition, one of the fundamental issues remains the lack of digital literacy and culture, and AI technology skills among healthcare professionals [68]. Currently, initial and continuing training programmes do not sufficiently integrate these technologies into the expertise that trainees (e.g., physicians, nurses) need to achieve to be authorised to practice. As reported in our study, without appropriate training, clinicians are unlikely to adopt in an appropriate way these technologies. Indeed, training is required to adapt provider protocols, administrative workflows, pathways, and business processes [67]. According to Mistry (2019), for such change to take place, healthcare professionals will need:1) to have access to education content enabling them to learn new skills as AI users and work differently; 2) to be able to train AI systems themselves for setting them up to perform specified tasks, which implies knowing what data to select and its quality; 3) to develop abilities to interpret AI outputs, including a solid understanding of its limitations and bounds of function; and 4) to know “how the system learns and what constitutes appropriate use, so that ethical norms are upheld and any introduction of biases is avoided” [67].

### Strengths and limitations

This study offers one of the first holistic and multilevel analyses of the complexity of the changes and transformations associated with the integration of AI technologies into clinical routine, beyond technical issues. It is also part of the few studies that go beyond looking at one single AI technology and delves into the organisational and systemic complexity of integrating multiple AI technologies concurrently.

However, the study has limitations. By its qualitative nature, it has a high level of internal validity, but the transferability (or generalisability) of its findings is limited to similar healthcare organisations and systems. In other contexts, it can increase the awareness of different stakeholders regarding the importance of taking better account of the sociotechnical dimension of AI. Healthcare organisations and systems can vary considerably, hence the importance of contextualising the results.

The number of interviewees (*n* = 29) is relatively low in view of the large number of AI technologies covered in this study. Although we made great efforts to include a wide range of stakeholders, several people were unable to participate due to the COVID-19 context. This is the case for women heading technology companies, whereas decision-makers, managers, and clinicians were unable to participate because of their direct involvement in the management of the pandemic. However, the people who participated, through their expertise and experience, provided us with rich data, necessary for a detailed understanding of the challenges of integrating AI in healthcare organisations and systems. The application of a rigorous research approach, guided by best methodological practices and an exhaustive theoretical framework, has reinforced the reliability of our results.

## Conclusions

AI in healthcare is still in its infancy. There are huge expectations that it will provide answers to major contemporary challenges in healthcare organisations and systems. This is reflected in the funding it receives from governments, but also in the interest of the financial and venture capital sector. The COVID-19 pandemic was a test case for AI, and it did not fully deliver. However, the pandemic has served as an accelerator for its experimentation, for example, through the relaxation of regulatory requirements and less resistance from some stakeholders. AI represents as much a logistical, psychological, cultural, and philosophical change, particularly in terms of what it could and should do in healthcare organisations and systems. It is a “new era” that requires a real critical examination to learn from the many past experiences with the digitalisation of healthcare organisations and systems. With AI, the nature, scale and complexity of the changes and transformations are at such a level and intensity that the implications could be profound for society. At present, little is known about how such an announced revolution may take shape and under what conditions. This study provides a unique learning base for analysing AI technologies in healthcare organisations and systems from a sociotechnical perspective using the NASSS framework. It adds to the existing literature and can better inform decision-making towards the judicious, responsible, and sustainable integration of these technologies in healthcare organisations and systems.

## Data Availability

The data that support the findings of this study are available from the corresponding author (HA) upon reasonable request. The data are not publicly available due to information that could compromise the privacy of the research participants.

## Appendix

**Table 5:** Illustrative quotes from participants organised around key themes of the NASSS framework

**Themes**	**Illustrative quotes from participants**
**The organisation**	Senior management “believes in the potential of AI in healthcare (…). Organisational leadership, that’s for sure. The City hospital also has a great reputation, even internationally. There’s a lot of publicity about AI at the City hospital. There are many articles about it. It certainly attracts talent (…). The culture encourages initiative. There are people who need the right environment to do AI (…). It’s a major factor.” [P25]
	“The City hospital has surgery; they have chemotherapy and radiation therapy. If you have a cancer treatment, you’re going to be treated at the City hospital for all three of this (…). In the USA, it’s very different where you might go to one location for radiation therapy, and then go over for chemotherapy, and then go to a hospital for surgery. And because of that, Canada is a very good place for us to develop this type of platform, because we have all these different treatments under one roof (…). It’s simpler for us to kind of connect the dots and to look at the big picture, rather than having to work with four different sites, with four different sets of data, to achieve the same thing.” [P3]
	“There are no mechanisms that require us to work together. Two things. I think that there are projects that are undertaken by too many departments working on the same projects. And that there are no mechanisms or some kind of transversal framework that forces us to work together. And there are no outcomes on which we are evaluated (…). And accountability, of course.” [P18]
	“Many AI technologies are developed and deployed in-house. At that point, any development of AI that comes from outside is going to bother them [The clinician-researchers who develop the technologies] (…). So, they will put obstacles in the way to prevent this external development being tested and proven internally, because they want their own to be promoted (…). There, they find themselves in ‘a conflict of interest’ as clinician-researchers (…).” [P1]
	“We received an invoice from the company saying: ‘You have so much data that we need a dedicated [Cloud] server’ (…). We ended up with a huge bill of CA$20,000 to CA$30,000/year. Management hadn’t initially foreseen this (…). In the end, we realised that it was going to cost an extra CA$20,000 to CA$30,000/year (…). With AI, we still don’t have a clear idea of how much it costs in the long term. And that’s the question of sustainability.” [P8]
**The condition(s) or illnesses**	“For patients, the main problem is waiting time (…). Of course, cancer is a progressive disease. So, that’s better outcomes because they’re going to be treated sooner (…). Just from the anxiety perspective, waiting is (…) one of the hardest parts of cancer, and just kind of sitting there and not knowing when you’ll be treated or not knowing what’s happening” [P3]
	“We asked the question: Can’t we use biomarker patterns to predict the response to a treatment or the therapeutic path that should be followed by the patient? (…). These aspects of predictive digital biomarkers can be derived from imaging data, genomic data, laboratory data, and textual data. So, the idea of these biomarkers is really to make precision medicine accessible (…). For example, to predict the response to a treatment. I’m not going to prescribe the wrong and expensive treatment for nothing. But above all, the disease will not progress while the patient is being prescribed the right treatment.” [P1]
**The technology or technologies**	“You know the ‘hype’ curve followed by the ‘valley of disillusionment’ for innovations (…). As soon as a company puts AI in its description, millions pour in, even though many of them are just advanced calculation systems that are not AI or Deep Learning (…). In the healthcare system, it’s still fancy.” [P28]
	“The head of IT security at an IT company is more aware of security issues than someone in a hospital system (…). It’s well known that the security systems in Quebec hospitals (…) are not well protected (…). A malicious company wanting to access health data can do so very easily (…). A company [like ours], which is subject to ISO [International organization for standardization] standards, has very strict quality standards with an audited security plan (…). The bar is much higher than in the hospital system, which is not ISO and has no penalties if there is a data leak (…). I can guarantee you that at [Company name], if there is an event of this kind or something goes wrong, either the company closes or there are employees who will be sacked.” [P1]
	“IT systems are disparate. Upgrade and interoperability challenges (…). Software upgrade cycles are very slow (…). The PACS [Picture archiving and communication system] we have is not even supported by the technology provider anymore. This creates problems because this data is necessary to use AI (…). For our archives, we still work with tapes (…). If it takes 3 or 4 months to retrieve the images, we’re not ready for that (…). It should be done in a few hours.” [P2]
	“There were several tools that were quite outdated (…). It’s well known everywhere (…). For example, to enter a patient into the information system, people did it manually: they wrote down the history, etc. Then, they scanned it in PDF format and entered it into the patient record (…). These are complex patients with co-morbidities, who are generally very sick. So, their record is going to be quite complex (…). They’re going to have twenty or more different documents attached [scanned in PDF format] with handwriting that we don’t understand from different clinicians (…). Plus, it’s not generally connected to other hospitals either (…). As a patient, if you go to one hospital and then another, there’s a good chance that you won’t have access to all your records.” [P22]
**The value proposition**	“There are different logics involved. Private companies want to promote their products and services. But the criteria for effectiveness are not the same on either side (…). We need to resolve this issue. It’s a problem that has always existed in public healthcare systems. In our healthcare system, we have all kinds of enthusiasm for launching projects (…). When the project reaches the end, we say: ‘Oh, we have to evaluate it’ (…). You must think about it in advance and ask evaluation questions: ‘What do we want from this technology?’” [P16]
	“If the hospitals are saving money, the government is saving money. And the main idea is just more trade, more patients can be treated. If typically, the healthcare system in Canada is treating one million cancer patients a year, we hope it can treat one hundred thousand more (…). We hope that our software can play a role in that.” [P3]
	“The company arrived at the City hospital. We told them that, in Oncology, we need something very specific, a sort of ‘widget’. But they’d like it to be a ‘shell opener’ because they can sell it for other things (…). It’s really this cultural schism. The generic solution and the ultra-specialised solution. The problem is that in medicine, there are lots of niche scenarios. There are lots of cancers. So, we need thousands of widgets. I’d love to have a technology that could diagnose all cancers, a ‘shell opener’. But that’s not possible. There are hundreds of cancers, each with its own biology and characteristics (…). The company’s rules just died in combat there.” [P2]
	“We receive demands [from companies] three or four times a week. It’s a huge volume (…). It’s the place where companies prioritise experimentation (…). One thing we’ve noticed is that we’re very passive and less proactive (…). For example, [We had clinical teams who] told us: ‘We don’t really like when you present companies to us because they don’t meet our needs. Can you go and find companies [that can meet the needs we’ve identified]?’ (…). It’s what we should be doing, and it’s very difficult (…). Often, we’d tell them: ‘You see, it is going to be free’. The hospital doesn’t have the resources.” [P8]
**The adopter system(s)**	“Yes, there is a danger (…) in the diagnosis (…). Yes, it’s 98% accurate. But there’s still the whole human relationship. And then, the clinician-patient decision about the action plan according to the life plan. I hope we don’t end up with: ‘Here’s the diagnosis. Here’s what we do’. On the contrary: ‘So, here’s the diagnosis, what do you want to do?” [P27]
	“It can increase the inequality gap (…). Not all people have access to these technologies at home (…). My relatives in the countryside don’t even have Internet at home, and the cellular network is only halfway through (…). [A family member] can’t wear a connected pacemaker because he/she doesn’t have internet (…). We must not forget the homeless person either, who will not necessarily use it, because it is a social reality to have access to this tool.” [P26]
	“We saw that a significant percentage of people said they were afraid of losing their jobs (…). People are not yet ready for change, they don’t have the skills to adopt the tools, to accept the change (…). But, I think, it’s our responsibility to develop these skills and to make these people aware of the change (…). To tell them: ‘It’s not the machine that will replace the clinician, but it’s the trained clinician who will replace the untrained clinician’.” [P5]
	“It’s not that they don’t want to (…). They’re very busy (…). They’re often stuck for 12 h a day. They need to be able to have at least 10% of their time devoted solely to the innovation project. But at the City hospital, clinicians are not paid to innovate. They’re told: ‘Are you going to add to your other tasks knowing that you don’t have the time and you’re not being paid to do it?’ (…). We’ll just end up with a few champions who want to do it.” [P4]
	“The data scientists didn’t understand the programming staff. The programming staff spoke a clinical-administrative language that the others didn’t understand. The latter didn’t know the reality on the ground. The ‘languages’ are not the same. *Vice-versa*, the programming staff didn’t understand everything the data scientists were saying either. It was a cultural shock. I was playing the role of ‘translator’ between these two worlds. I understand the AI (without being an expert) and I understand the clinical and administrative aspects.” [P14]
	“Clinicians need to know what kind of data the model has trained on. For example, if the model doesn’t take ethnicity into account in its training data and a new publication, or guideline, says that when you do lung screening, you must look at the patient’s ethnicity, because, for example, [ethnicity] patients have more X mutations (…). As a clinician, if I know that the model only looks at imaging and pathology but not at ethnicity, then I know that my model might not be good anymore.” [P1]
**Embedding and adaptation over time**	“There are also all the regulatory and procurement challenges, with the rigid infrastructure, which reduces our technological responsiveness. It’s not so easy to be responsive, because the laws are not agile enough. You add to that the certification from Health Canada if the technology is going to change.” [P14]
	“This is what happens with [one of our technology providers] (…). We’re completely tied up and dependent on their solution for the rest of the time (…). Updating is expensive. Access to data is expensive. Even to access our own data, we must pay. These companies ‘chain and lock’ hospitals.” [P8]
**The wider system**	“Zero risk does not exist. If I have a manual process that has an error rate of 30% and I reduce it to an error rate of 10% thanks to technology. I’d have 10 patients who wouldn’t be happy (…). Patients need to realise that, unfortunately, the diagnosis of terminal cancer is perhaps the fault of the healthcare system. The patient has been waiting a year for a diagnosis, what’s going on? Yes, errors do happen, unfortunately (…). A bit like when a plane crashes. An investigation is conducted to (…) correct these failures.” [P1]
	“They were obliged to use a partnership contract, because it spared them the whole process and the requirements of a tender with the MSSS, especially in terms of being able to integrate innovations quickly into the City hospital (…). At the same time, a partnership contract is restrictive in terms of being able, for example, to integrate certain technologies into the clinical routine, fund them and sustain them, etc. (…). The slightest financial contribution is subject to the rules of the tender and the Act on contracting by public bodies.” [P11]
	“They were oriented towards ‘capital equipment’. When you told them that the service was by ‘subscription’, they’d say: ‘But we don’t have a way of budgeting for that’. You must tell them how much it costs (…). In Quebec, we’re stuck with the lowest bidder tenders (…). It’s not the same as saying you’re signing a contract on a ‘Software as a Service’ basis, because everything always must be up to date. And yes, there are recurring costs, because it’s a solution as a service that doesn’t have the notion of ownership, where you buy the code, and everything belongs to you.” [P28]
	“Procurement processes are not really tailored to digital technologies. Especially not the procurement processes that are designed for SMEs that commercialise innovative technologies. The procurement process is designed to buy from [names of major technology companies]. But the small company, with a technology that would lead to, for example, half a million dollars a year (…), [it can hardly have contracts under the current conditions]. [P24]
	“Regarding data leakage or hacking (…). Legally, we are asking the company to take responsibility for this (…). There is no way of guaranteeing security, and the event could happen (…). From a negotiation perspective, it’s always very difficult, because there aren’t many people willing to take responsibility for major data issues (…). It involves large amounts of money if the organisations are held liable. This is what makes it so difficult to integrate these technologies into healthcare organisations.” [P6]
	“It’s not yet clear-cut (…). Will the company have medico-legal responsibility? No. They want to privatise profits and socialise responsibilities (…). The companies are not stupid (…). They are going to present their AIs as triage tools (…). They’re going to say: ‘We’re on the triage level, we’re raising the flag. You deal with the rest. We’ll detect the stroke, we’ll notify the neuroradiologist (…). It’s up to you to confirm the diagnosis. So, we wash our hands of it’. But when you dig a little deeper, the triage software has detected a vessel filling defect. Basically, it says: ‘There’s a stroke there, or a thrombosis there’ (…). Now, we’re no longer in triage. We’re in classification (…). But from a medico-legal point of view, it’s better to say triage, so it’s not diagnosis.” [P2]
	“Coming back to cost/benefit. For example, reduction in emergency room admissions, reduction in 30-day re-hospitalisations (…). We can monetise at the MSSS (…). Quality of life is the responsibility of other ministries. How do you convince the MSSS, for example, that if I gain 4 points in QALY, that’s CA$40,000 per patient? The MSSS is going to say: ‘That’s not in my budget, so why should I fund you?’.” [P14]
	“[For example, in] AI and cancer imaging (…). We’ll need to obtain multicentre data from several Quebec or Canadian hospitals. We need data sets containing tens of thousands of patients. The City hospital cohorts will not be enough. Articles are appearing, published in Nature Journal. There are two hundred thousand patients from multicentre studies (…). No modern university hospital has these sample sizes. We need to move towards federated training.” [P2]

## Abbreviations

AI: Artificial intelligence
CA$: Canadian Dollar
COVID-19: Coronavirus Disease 2019
DGTI: Quebec’s Ministry of Health and Social Services Information Technology Division
EHR: Electronic Health Record
ISO: International Organization for Standardization
IT: Information Technology
LCOP: Act on Contracting by Public Bodies
MRI: Magnetic Resonance Imaging
MSSS: Quebec’s Ministry of Health and Social Services
NASSS: Non-Adoption, Abandonment, Scale-up, Spread, Sustainability
PACS: Picture Archiving and Communication System
PDF: Portable Document Format
QALY: Quality-Adjusted Life Year
SaaS: Software as a Service
SaMD: Software as a Medical Device
SU/SMEs: Start-ups and Small- and Medium-sized Enterprises
US$: United States Dollar
USA: United States of America
VBP: Value-Based Procurement
